# Non-alcoholic Wernicke’s Encephalopathy Masquerading As CNS Relapse of Acute Myeloid Leukemia

**DOI:** 10.7759/cureus.61184

**Published:** 2024-05-27

**Authors:** Ammad J Chaudhary, Katherine M Joyce, Kamran Haq, Muhammad Hamza Qureshi, Vijayalakshmi Donthireddy

**Affiliations:** 1 Internal Medicine, Henry Ford Health System, Detroit, USA; 2 Emergency Medicine/Internal Medicine/Critical Care, Henry Ford Health System, Detroit, USA; 3 Internal Medicine, Mayo Hospital, Lahore, PAK; 4 Hematology/Oncology, Henry Ford Health Sysytem, Detroit, USA

**Keywords:** extended hospitalization stay, neurological signs and symptoms, chemotherapy induced nutritional deficiency, thiamine or vitamin b1 deficiency, non-alcoholic wernicke's encephalopathy

## Abstract

While Wernicke's encephalopathy (WE) is mostly caused by thiamine deficiency secondary to chronic alcohol use, other conditions that may affect one’s nutritional status, such as bariatric surgery, hyperemesis gravidarum, chronic gastrointestinal disease, HIV/AIDS, and certain malignancies, may also lead to this outcome. We are discussing one such case, WE, in a young man with acute myeloid leukemia (AML) who underwent chemotherapy. The patient presented with blurred vision, paresthesia, weakness, and vomiting. Although he denied alcohol abuse, his symptoms, physical exam findings, and MRI results were consistent with WE. Treatment with thiamine resulted in a significant improvement in his visual disturbances and mental status. The authors highlight the importance of recognizing WE in non-alcoholic patients, particularly those undergoing prolonged hospitalization and chemotherapy, as nutritional deficiencies can develop. They recommend thiamine supplementation for patients receiving chemotherapy and those with poor oral intake. The case underscores the need for high clinical suspicion and early intervention in atypical cases of WE.

## Introduction

Wernicke's encephalopathy (WE) is an acute neurological condition caused by a thiamine deficiency (vitamin B1). WE is classically associated with alcohol use disorder, but patients who do not consume alcohol can also present with this condition. Many WE cases have been discovered post-mortem and significant literature suggests that the disease process is underdiagnosed [1]. Due to the large number of WE patients presenting with a history of chronic alcoholism, there is a low clinical suspicion of WE in patients who do not present with such or have atypical case presentations [2]. In cancer patients, WE is caused by a combination of decreased availability as a result of malnutrition and anorexia secondary to the disease and treatment and accelerated usage of thiamine in metabolically hyperactive tumor cells [3]. If not identified and treated urgently, WE can progress to Wernicke-Korsakoff syndrome (WKS), resulting in permanent neurological damage or death. WE characteristically presents in patients with alcohol use disorder as a triad of neurological symptoms consisting of changes in mental status, ataxic gait, and ophthalmoplegia/nystagmus. While this triad may be “classic,” it is absent in most patients [4]. There have been case reports of Wernicke’s in patients diagnosed with cancer, many of whom are actively undergoing chemotherapy and/or using TPN. We present the case of a young man with a history of acute myeloid leukemia (AML) who required two courses of back-to-back induction chemotherapy and no history of significant alcohol abuse. He presented with acute neurological and ophthalmic symptoms with remarkable improvement after parental administration of thiamine.

## Case presentation

A patient in his early 20s with a known history of AML status-post-two rounds of chemotherapy initiated three months prior-presented to the Emergency Department (ED) with blurred vision, bilateral lower extremity paresthesia, weakness, fatigue, and vomiting. He had been treated with 7+3 cytarabine/daunorubicin induction therapy (seven days of cytarabine followed by three days of daunorubicin), followed by 14 days of midostaurin. A repeat bone marrow biopsy post-induction therapy revealed 42% blasts and refractory FMS-like tyrosine kinase 3 (FLT3)-positive AML on molecular testing. Consequently, at day 30 from the initiation of the induction therapy (seven days after completion of the first treatment), a second induction course was commenced for refractory AML with a six-day course of FLAG-IDA (fludarabine, high-dose cytarabine, idarubicin, and granulocyte-colony stimulating factor). One month after the second completed treatment, the bone marrow biopsy showed 1% blasts. The inpatient stay was 29 days for the first round of induction therapy, followed by 24 days inpatient for the second round of induction therapy. 

He presented to the ED 29 days after discharge from his second induction therapy, approximately three months after beginning his first round of induction chemotherapy. In the ED, he was lethargic, with a physical exam revealing gait imbalance and horizontal nystagmus on both the right and left gazes. He reported smoking marijuana one to two times a week but denied alcohol consumption or other substance use. He endorsed having a poor appetite but denied weight loss, with a BMI of 29 kg/m^2^ on presentation. Physical assessment revealed no signs of overt malnourishment, such as temporal muscle wasting. Laboratory data was significant for a low blood urea nitrogen level of 4 mg/dL with a normal estimated glomerular filtration rate and creatinine, a high-normal albumin concentration, a magnesium level of 1.8 mEq/L, and mild hypokalemia at 3.2 mEq/L. However, these laboratory abnormalities did not explain his symptoms' severity. Point-of-care glucose was within the reference range at 137 mg/dL. Computed tomography (CT) of the head without IV contrast was unrevealing and showed no acute intracranial processes. There was a concern for infection, as he had leukocytosis (12.7 K/µL) with an absolute neutrophilic predominance. Urine toxicology was positive for opiates, which were given in the ED before urine collection, and cannabis. His symptoms did not improve with fluids or antiemetics.

**Figure 1:**
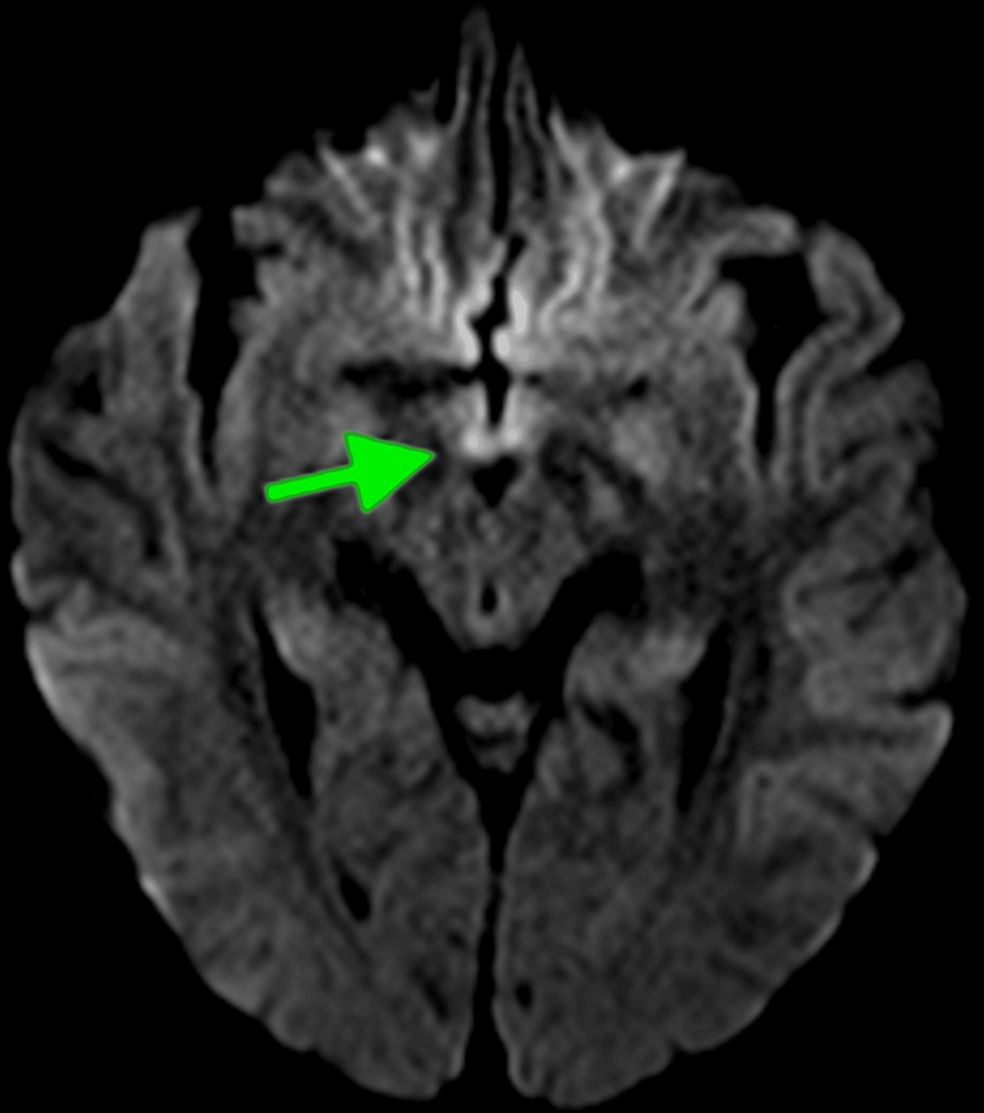
MRI brain with T2 imaging revealing edematous mammillary bodies (green arrow)

**Figure 2:**
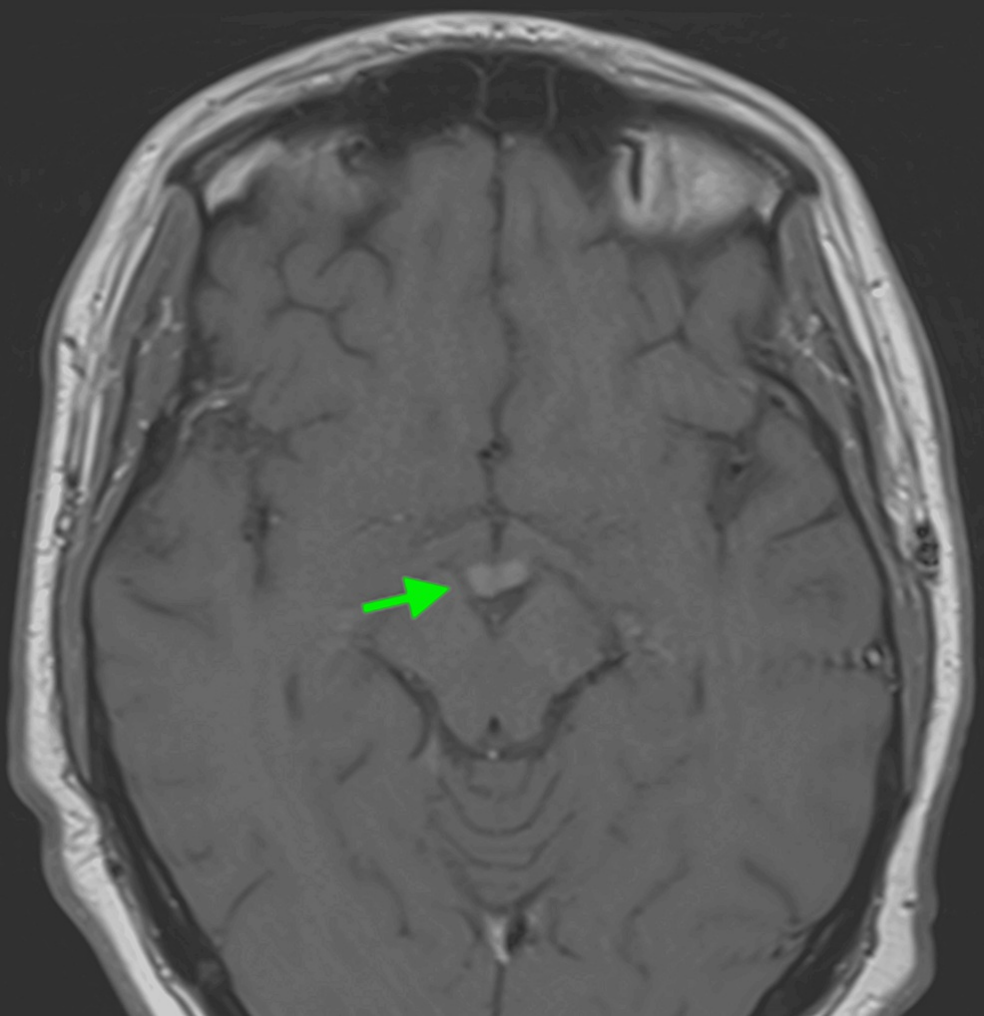
MRI brain with FLAIR imaging revealing edematous mammillary bodies (green arrow)

Magnetic resonance imaging (MRI) of the brain was done, which revealed edematous mammillary bodies with increased signaling (green arrow) on T2 (Figure 1) and FLAIR imaging (Figure 2). While awaiting a lumbar puncture in the interventional radiology suite to explore possible central nervous system (CNS) leukemia dissemination, MRI findings and subsequent consultations with neurology and ophthalmology favored WE higher on the differential.

He was started on IV thiamine (500mg) three times daily for 48 hours, followed by 250 mg IV thiamine daily for four days. His thiamine levels after the first dose of 500 mg IV thiamine were 101 nmol/L. His subsequent levels were 148 nmol/L and 153 nmol/L on days 2 and 3 of thiamine supplementation, respectively. He was then transitioned to oral daily thiamine 100mg. Upon administration of thiamine, the patient's visual disturbances and mental status improved significantly in less than 24 hours. Paresthesia and gait imbalance did persist, which is expected and consistent with current literature reports [5]. He was discharged after a six-day stay with significant improvement in his overall condition. He was discharged home on daily supplementation of thiamine with a one-month follow-up. He was advised to continue using thiamine until he receives the bone marrow transplant.

During follow-up, the patient showed significant improvement in his symptoms, although weakness persisted. Over time, his gait disturbance and weakness gradually improved, with near-complete resolution reported during the stem cell transplant follow-up one month after discharge from the initial ED presentation. The patient continues to take a thiamine supplement and is doing well overall.

## Discussion

Thiamine is a coenzyme for many important enzyme complexes involved in carbohydrate metabolism and, therefore, plays a crucial role in the process. A lack of thiamine can lead to inadequate energy production and an accumulation of metabolites that can cause damage to high-energy-dependent tissues, particularly central and peripheral neurons [6]. The presenting symptoms of WE due to thiamine deficiency are mainly neurological, manifesting as a triad of mental status changes, nystagmus, and ataxia [7]. However, accurate diagnosis can be challenging as presentations can vary depending on the underlying cause of WE, with alcoholic patients presenting predominately with cerebellar signs and nonalcoholic patients presenting with predominant ocular signs [8].

MRI can aid in the diagnosis of WE in cases where the diagnosis remains questionable based on clinical presentation alone and can delineate between alcoholic and nonalcoholic WE. Imaging studies of WE typically show involvement of the periventricular and periaqueductal regions, along with the cerebellum, thalamus, and mammillary bodies, with alcoholic patients exhibiting more pronounced cortical and subcortical atrophy compared to nonalcoholic WE patients [8].

The clinical impression was initially considered to be AML extension into the CNS, given the patient's clinical history of mild leukocytosis, elevated lactate dehydrogenase (LDH), and a known FLT3-TKD mutation [9,10]. Evidence of AML spreading to the CNS can involve leukemic cells within the cerebrospinal fluid, meningeal infiltrates, or a solid CNS mass. Limited literature is available on the specific radiographic findings of CNS AML involvement, especially in adult patients. One study investigated the neuroimaging results of childhood AML with CNS involvement and reported that intracranial leukemic tumors and meningeal enhancements were rarely seen in their sample [11]. There do not appear to be any particular brain regions or MRI findings particular to AML with CNS involvement, yet further studies are needed to confirm this observation. Although the LP could not be completed, we would not have expected this patient's symptoms to improve substantially with thiamine alone if blasts were present in the CNS.

When looking at a sample of cancer patients with nonalcoholic WKS, Isenberg et al. suggested diagnosis should be made using the operational criteria versus the traditional “Wernicke's triad,” listed above, due to its limiting presentation [12]. The operational criteria proposed by Caine et al. allow for the diagnosis of WE when at least two of the following are present: nutritional deficiency, ocular signs, cerebral signs, and either altered mental status or impaired memory [13]. Moreover, Isenberg et al. noted that those diagnosed with WE were not underweight and had normal serum concentrations of B12 and folate despite being thiamine deficient. Our patient's condition improved significantly with just one dose of thiamine, which further favored the diagnosis of WE. Prior research on similar cases has indicated this diagnosis is appropriate based on the clinical picture and one of the following: MRI findings, clinical improvement, and/or thiamine levels. Our patient still had gait disturbances following the administration of thiamine; however, this is not entirely unusual, as it has been reported that patients typically have delayed recovery from gait ataxia secondary to thiamine deficiency [14].

Many conditions are associated with the cause of WE, which essentially result in a nutritional deficiency in thiamine either through decreased intake, impaired absorption, or increased cellular demand for thiamine. It is well documented that WE can occur in patients with chronic alcoholism due to poor nutrient intake and absorption. However, the literature also reports other less common etiologies of WE, such as bariatric surgery, hyperemesis gravidarum, chronic gastrointestinal disease, HIV/AIDS, cannabis use and certain malignancies [7,15]. Hematological and gastrointestinal cancers have been reported as carrying the highest risk among malignancies for developing a thiamine deficiency due to accelerated consumption by tumor cells and decreased absorption, respectively [3]. In addition to hematologic malignancy, our patient had a poor diet during his inpatient stay. While his caloric needs seemed to be met, the nutrient density of his diet was poor. Patients with a prolonged inpatient stay can easily become deficient in thiamine if not supplemented properly with a well-balanced diet, as human thiamine storages only last two to three weeks [16].

Although treating thiamine deficiency may seem simple enough, nutritional supplementation has risks. In patients with prolonged nutritional deficiency, caution should be taken when nutritional replenishment therapy is begun, given the risk of developing re-feeding syndrome, which can manifest as electrolyte derangements as well as a further decrease in thiamine stores if thiamine is not supplemented before indicating proper nutrition [17]. Otherwise, thiamine administration is generally considered safe. It is also necessary to consider magnesium levels in patients with disease processes resulting from thiamine deficiency. Magnesium allows for proper utilization of thiamine, with hypomagnesemia indicated in cases of WE refractory to thiamine supplementation [18]. Hypomagnesemia was not a significant concern during our patient's presentation, as he responded well to the thiamine supplementation. This case highlights the importance of maintaining a high clinical suspicion for WE in atypical patients.

## Conclusions

Thiamine is vital in energy production, and it's essential to recognize that thiamine deficiency leading to WE is not just seen in chronic alcohol users. Other factors, like poor nutrition, cancer, and chemotherapy, can also cause it. This is especially important for cancer patients who spend a lot of time in the hospital and get chemotherapy. Clinicians should maintain a high clinical suspicion for WE in patients with a history of malignancies, particularly those with prolonged hospital stays receiving chemotherapy who present with acute neurological symptoms, to prevent the long-term consequences of untreated WE and ensure a better prognosis for patients.
